# Case study of the automation options and decisions made in implementing a high-throughput cell based screen using the FLIPR™

**DOI:** 10.1155/S1463924600000213

**Published:** 2000

**Authors:** Derek J. Hook

**Affiliations:** 1 951 East Rocky Mouth Lane Draper UT 84020 USA; 2 Biomolecular Screening NPS Pharmaceuticals 420 Chipeta Way Salt Lake City UT 84108 USA

## Abstract

This case study examines the automation and process change options available to emerging discovery/development stage pharmaceutical companies when considering implementing sophisticated high-throughput screens. Generally there are both financial and personnel constraints that have to be addressed when implementing state-of-the-art screening technology in smaller companies which generally are not as significant as in large pharmaceutical companies. When NPS Pharmaceuticals considered installing a Molecular Devices FLIPR™ for high-throughput cell based screening it became clear that, to make the best decision, the whole screening process at NPS Pharmaceuticals from screen development and validation, tissue culture, compound distribution, data handling and screening had to be re-examined to see what automation options were possible and which, if any, made sense to implement. Large scale automated systems were not considered due to their cost and the lack of in-house engineering infrastructure to support such systems. The current trend towards workstation based laboratory automation suggested that a minimalist approach to laboratory automation, coupled with improved understanding of the physical process of screening, would yield the best approach. Better understanding of the work flow within the Biomolecular Screening team enabled the group to optimize the process and decide what support equipment was needed. To install the FLIPR™, train users, set up the tissue culture protocols for cell supply, establish high-throughput screening database protocols, integrate compound distribution and re-supply and validate the pharmacology on four cell based screens took the team 3 months. The integration of the screening team at the primary, secondary and tertiary screening stages of the target discovery project teams at NPS has enabled us to incorporate minimal automation into the Biomolecular Screening Group whilst retaining an enriching work environment. This is reflected in our current consistent throughput of 64 96-well microplates per day on the FLIPR™, a figure that is comparable with that achieved within most major pharmaceutical companies. This case study suggests that process optimization coupled with modern stand alone automated workstations can achieve significant throughput in a resource constrained environment. Significantly greater throughput could be achieved by coupling the process improvement techniques described above with 384-well microplate technology.


Journal of Automated Methods & Management in Chemistry, Vol. 22, No. 5 (September–October 2000) pp. 139–142

### Case study of the automation options and decisions made in implementing a high-throughput cell based screen using the FLIPR™

**Derek J. Hook**

*951 East Rocky Mouth Lane, Draper, UT 84020, USA. Former Director, Biomolecular Screening, NPS Pharmaceuticals, 420 Chipeta Way, Salt Lake City, UT 84108, USA*

*This case study examines the automation and process change options available to emerging discovery/development stage pharmaceutical companies when considering implementing sophisticated high-throughput screens. Generally there are both financial and personnel constraints that have to be addressed when implementing state-of-the-art screening technology in smaller companies which generally are not as significant as in large pharmaceutical companies. When NPS Pharmaceuticals considered installing a Molecular Devices FLIPR™ for high-throughput cell based screening it became clear that, to make the best decision, the whole screening process at NPS Pharmaceuticals from screen development and validation, tissue culture, compound distribution, data handling and screening had to be re-examined to see what automation options were possible and which, if any, made sense to implement. Large scale automated systems were not considered due to their cost and the lack of in-house engineering infrastructure to support such systems. The current trend towards workstation based laboratory automation suggested that a minimalist approach to laboratory automation, coupled with improved understanding of the physical process of screening, would yield the best approach. Better understanding of the work flow within the Biomolecular Screening team enabled the group to optimize the process and decide what support equipment was needed. To install the FLIPR™, train users, set up the tissue culture protocols for cell supply, establish high-throughput screening database protocols, integrate compound distribution and re-supply and validate the pharmacology on four cell based screens took the team 3 months. The integration of the screening team at the primary, secondary and tertiary screening stages of the target discovery project teams at NPS has enabled us to incorporate minimal automation into the Biomolecular Screening Group whilst retaining an enriching work environment. This is reflected in our current consistent throughput of 64 96-well microplates per day on the FLIPR™, a figure that is comparable with that achieved within most major pharmaceutical companies. This case study suggests that process optimization coupled with modern stand alone automated workstations can achieve significant throughput in a resource constrained environment. Significantly greater throughput could be achieved by coupling the process improvement techniques described above with 384-well microplate technology.*

### Introduction

High-throughput screens at NPS Pharmaceuticals include cell based assays that are designed to discover agonists and antagonists of G-protein coupled receptors (GPCRs). They are based on the ability to measure intracellular calcium concentration changes as a result of the secondary signalling responses of these GPCRs to test substances. The response is monitored through fluorescent changes of the intracellular dye Fluo-3 as a response to changing calcium concentration.

### Experimental

Historically, the equipment used was an 8-channel fluorometer with integrated liquid handling, capable of reading one column (eight wells) of a 96-well microplate every 5 minutes. This equipment was constructed in-house from commercially available components, and consisted of a Hamilton MPH2200 8-probe liquid handler (Hamilton Co., Reno, NV), an X–Y stage to move the microplate across the deck of the liquid handler, and an 8-light pipe fibre optic bundle underneath the microplate to provide light paths for both the excitation light and to feed the emission light to a single photomultiplier tube (PMT) multiplexed to the 8-fibre optic bundle using a rotating scanning mirror. The photomultiplier signal was detected by a signal processing unit, the output of which was digitized and stored in a computer. All the control software for the X–Y stage and the motor controlling the mirror scanning the PMT were written in-house and these motors were controlled by a PC responsible for digitizing and collecting the signal data. A separate PC with a handshake via an I/O port controlled the Hamilton liquid handler and initiated the liquid handling operations. All the equipment was contained within a darkroom to exclude ambient light from the test plate. Typically the time for analysing a 96-well microplate was about 60 minutes.

After purchase of the FLIPR™ from Molecular Devices, the equipment configuration for the assay consisted of the FLIPR™ I (Molecular Devices, Sunnyvale CA), a Skatron model 301 ScanWash/ScanStack plate washer (Molecular Devices, Sunnyvale, CA), a Hamilton MPH2200 16 probe liquid handler (Hamilton Co., Reno, NV) and a Forma model 310 tissue culture incubator (Forma Scientific/Savant Instruments, Marietta, OH). All these units were contained in ambient laboratory conditions (no darkroom). Typically the time to analyze a 96-well microplate was about 5 minutes. Bioinformatics was handled by ActivityBase (ID Business Solutions, Emeryville,CA).

*Process flow with in-house fluorometer system*

1 week before assay:

plate out HEK-293 cells containing the human calcium receptor, grow at 37°C for 7 days, replacing medium every day.

On the day of assay:

- Cell loading:
  (1) add buffer containing Fluo-3 to the microplate,
  (2) incubate microplate in the dark for 60 minutes,
  (3) remove loading buffer,
  (4) add test buffer.
- Test compound plate preparation:
  (1) whilst cell assay plate is being loaded with fluorophore, place microplate on Hamilton liquid handler,
  (2) initiate addition of buffer from reservoir on Hamilton,
  (3) mix buffer in test plate wells, washing between additions.
- Assay:
  (1) place microplate containing cells loaded with dye on X–Y stage on Hamilton,
  (2) initiate start sequence on system (built in 15 second delay),
  (3) exit darkroom and close door,
  (4) repeat after microplate run is completed.

*Process flow with FLIPR™ I system*

24 hour prior to assay:

(1) plate cells at validated density from flasks,

(2) grow overnight at 37°C in tissue culture area,

(3) transfer assay flasks to screening tissue culture incubator.

On the day of assay:

- Test compound plate preparation:
  (1) load 16 plates on to 16-probe Hamilton liquid handler,
  (2) load buffer reservoirs,
  (3) initiate Hamilton addition program,
  (4) repeat four times.
- Cell loading:
  (1) load first plate with Fluo-3,
  (2) put in dark area,
  (3) 5 minutes later, load second plate with Fluo-3,
  (4) put in dark area,
  (5) repeat every 5 minutes for total plate run on that day,
  (6) after 30 minutes, remove 1st plate,
  (7) put plate on Skatron washer,
  (8) run Skatron washer to wash off loading buffer and to add final test buffer,
  (9) repeat every 5 minutes.
- FLIPR™ assay:
  (1) open tip loading hatch,
  (2) load new set of tips,
  (3) open FLIPR™ microplate drawer,
  (4) place assay plate in centre location of FLIPR™ loading tray.
  (5) place compound plate in right hand location in FLIPR™ loading tray,
  (6) place challenge plate in left hand location in FLIPR™ loading tray,
  (7) shut FLIPR™ microplate drawer,
  (8) open new data file,
  (9) initiate test sequence,
  (10) shut data file.

*Process flow for secondary and tertiary assays:*

(1) *Cuvette-based.* Prepare buffers, load cells, put in single cuvette fluorimeter, add test compound, measure response, 5 minute assay. Automated data capture of curve, manual curve analysis. Repeat for next concentration of agonist, antagonist. Throughput was limited. 25 compounds with an 8 point concentration curve required 6 days of testing and 30 hours of machine time.
(2) *FLIPR™ microplate based.* 8 point curve run on 25 compounds. Preparation, loading, testing 1 day, 30 minutes of machine time.

### Results and discussion

The results for the productivity and reduction in waste in the Tissue Culture Group were remarkable. Effort expended in validating correct plating densities, expansion of cells in tissue culture flasks instead of microplates and plating cells on demand 24 hours before the assay resulted in a reduction of wasted plates from about 50% of plates with the old process and instruments to less than 5% of plates. At a throughput of 64 plates per day, over a 4 month period this potentially saved about 10 000 plates from being wasted, about a $50 000 saving in materials cost, not counting downtime. This also allowed greater flexibility in assay schedule, since cells could be maintained in tissue culture flasks up to 24 hours prior to the assay when a commitment to the cell line for the next day would be made. If priorities changed, then cell lines could be switched with about 24 hours notice, impossible if a one week long cell growth cycle in microplates had continued.

The results on the throughput of the assay was also remarkable. Prior to the installation of the FLIPR™, throughput was limited to about two runs of eight plates per day. Even though this increased slightly when the second home built machine was operational, continued problems with maintaining the unit operational never achieved the expected productivity gains. Installation of the FLIPR™ when fully operational resulted in four test cell lines and the control wild type cell line being run per week, a total throughput of 320 plates per week, a 20-fold improvement. This was maintained for the 5–6 month operation of the unit for these screens.

The major problems encountered in attempting to upgrade the in-house 8-channel fluorometers included machine serving difficulties, lack of in-house engineering expertise, space requirements, obsolete and unobtainable spare parts, differences in fibre optic bundles between machines, lack of reproducibility in obtaining data between machines, unreliability of the 16-probe upgrade to one machines and requirements for a separate darkroom for each machine.

After the problems encountered in attempting to upgrade the in-house fluorometer systems became extreme, we evaluated the FLIPR™ I at Molecular Devices' facilities in Sunnyvale. These tests, which were conducted over two days, convinced us that replacement of the in-house systems with a FLIPR™ I would be the most cost effective solution because the results were reproducible, the limit of detection of response was lower, the quality of the data was better and the throughput potential was high. A purchase decision was made in June 1998, the machine was delivered and installed by mid-July and training of the users completed by the end of July 1998. This included the time necessary to make mechanical changes to the room and to establish new electrical and cooling water services for the FLIPR™.

During this period we developed new process protocols to allow operation for four out of five days per week. Included in this process was an evaluation of what automation or robotic systems should be implemented. Constraining our decision was the realization that we were dealing with a cell based system that required dye loading and maintenance of physiological conditions throughout the assay. Together with the non-robotic friendliness of the FLIPR™ I system, a lack of in-house engineering support and a need to have the system fully operational as quickly as possible made us decide not to integrate the FLIPR™ I into a robotic system. We did decide to use existing liquid handling workstations to handle plate preparation tasks, and purchased a Skatron plate washer to make the dye loading and microplate washing steps more effective. The plate transfer steps were done manually. We started at relatively low throughput, and gradually increased plate throughput, with the operators finally feeling comfortable at 64 plates per day. One method used to achieve this throughput was to use two operators per day, one assisting in buffer and test plate preparation and the other running the FLIPR™.

Screen validation began at the end of July 1998 and we were running 32 plates per day by the end of November 1998, with bioinformatics protocols in place. By the end of December 1998 we had increased throughput to 48 plates per day and had started writing the secondary and tertiary screening protocols. By the end of February 1999 we had five screens running at 64 plates per day, with all bioinformatics protocols operating. Usually we double up on screens on one day per week, so the daily throughput achieved here was in fact 128 plates per day or 10 000 samples per day. By the end of August 1999 all five screens had finished the entire NPS compound deck.

Although it is easy to quantify the throughput of the assay, and identify a productivity improvement of 12–20-fold, this was achieved only by integrating the workflow of the screening, tissue culture, bioinformatics and compound distribution groups. During the initial screen validation period, three essential processes took place. The first of these was the establishment of tissue culture protocols for the growth of the recombinant cell lines in both flasks and microplates. One important parameter that was established through experimentation was the plating density required for a viable assay in the FLIPR™. Once this was established the plating density required at 24 hours prior to assay was established, and from this was derived the number of T-125 flasks and seeding/splitting strategy needed to provide the total number of cells to plate 64 microplates for next-day screening. The second of the processes was the establishment of a database protocol for the primary screening assay. The protocol for the first screen took the longest. Protocols for later screens were essentially copy, cut and paste operations from this initial screen protocol. The first protocol was written in parallel with the development of the corresponding cell line tissue culture protocols, and so was ready when screening was initiated. The third process was the validation of the pharmacology of the screen and the assay conditions. Variables that were optimized included dye loading times and dye concentration, the buffer type and composition, addition or not of dye leakage inhibitors and controls and standards and their concentration on the microplate.

Another measure of productivity gain is shown by indication that 7 to 10 FTEs were used in June 1997 for primary, secondary and tertiary screening with the old in-house rigs and, in comparison, after March 1999, only 4 FTEs were needed for the same or greater amount of screening in the primary, secondary and tertiary phases.

There were other benefits from the FLIPR™ based assay with limited automation. The instrument itself was always accessible manually and not integrated as part of a large automated system. This made it easy for small runs of plates during screen development to be accommodated. Also the increased data output enabled responses to medicinal chemistry SAR to be made more rapidly by the screening group for the project team. In addition, the assays run each day on the machine could be scheduled more flexibly, improving response time to project needs and demands.

These process and hardware changes have reduced waste, decreased downtime and ultimately have decreased the cost per compound screened. This was accomplished with minimal cost—two new stand alone workstations (the FLIPR™ and the Skatron plate washer). Savings were achieved by reuse of the Hamilton liquid handlers that were part of the old in-house screening rigs as compound plate preparation units.

### Summary

Before making a commitment to a large scale tightly integrated system, smaller companies need to consider the need for rapid screen implementation, the availability of core engineering resources and capital costs as key issues. It may be that alternative process improvement steps that can be done at no cost and use loosely integrated automated workstations as alternatives provide a more flexible solution to throughput improvements. As in the case of NPS's cell based assays, throughput comparable to or exceeding that attainable in major pharmaceutical companies with greater human and financial resources was achieved in a sustainable manner. A whole compound deck of 100 000 compounds could be screened within 6 weeks using the processes and systems described here. As plate densities increase from 96-well to 384-well plates for FLIPR™ type cell based assays, use of the FLIPR™ II in a similar minimally automated system could yield throughputs at the threshold of 100 000 samples/week in cell based functional assays.

### Acknowledgements

I would like to acknowledge: the high throughput screening group currently at NPS Pharmaceuticals, Linda Artman – Screening team leader, Noelle Smith, Joanna Johnston, Will Heaton; the Tissue Culture Core Facility with Pam Jacobson, Natasha Lloyd and Gina Ramoz-Leslie; Jill Petik who assisted with Hamilton programming; former screening group members Matt Peach, Rob Dutcher, Kathleen Hamer and Steve Kubala; Doug Fairbanks from compound distribution; Charlie Hussey from bioinformatics; Ben Hung who also assisted in screening; and two of our consultants in project management and process flow, Ernie Nielsen and Steve Shaha. All these people contributed as a team to optimizing the screening process at NPS Pharmaceuticals and generated much of the data given in this paper.

### Trademarks

FLIPR is a trademark of Molecular Devices Corporation, SkanWash and SkanStak are trademarks of Skatron Corporation, AcivityBase is a trademark of ID Business Solutions.

